# Systematic review of outcome measures in trials of pediatric anaphylaxis treatment

**DOI:** 10.1186/1471-2431-14-158

**Published:** 2014-06-20

**Authors:** Tamar Rubin, Jacqueline Clayton, Denise Adams, Hsing Jou, Sunita Vohra

**Affiliations:** 1 Department of Pediatrics, University of Alberta, Edmonton, Canada; 2 CARE Program, University of Alberta, Edmonton, Canada; 3 Department of Public Health Sciences, University of Alberta, Edmonton, Canada

## Abstract

**Background:**

Considerable heterogeneity has been observed in the selection and reporting of disease-specific pediatric outcome measures in randomized controlled trials (RCTs). This makes interpretation of results and comparison across trials challenging. Outcome measures in pediatric anaphylaxis trials have never previously been systematically assessed. This systematic review (SR) identified and assessed outcome measures used in RCTs of anaphylaxis treatment in children. As a secondary objective, this SR assessed the evidence for current treatment modalities for anaphylaxis in the pediatric population.

**Methods:**

We searched MEDLINE, EMBASE, The Cochrane Library, Cochrane Central Register of Controlled Trials (CENTRAL), and CINAHL from 2001 until December 2012. We also searched websites listing ongoing trials. We included randomized and controlled trials of anaphylaxis treatment in patients 0–18 years of age. Two authors independently assessed articles for inclusion.

**Results:**

No published studies fulfilled the inclusion criteria.

**Conclusions:**

There is an alarming absence of RCTs evaluating the treatments for anaphylaxis in children. High quality studies are needed and are possible to design, despite the severe and acute nature of this condition. Consensus about the selection and validation of appropriate outcome measures will enhance the quality of research and improve the care of children with anaphylaxis.

**Trial registration:**

CRD42012002685

## Background

RCTs are the gold standard for clinical treatment efficacy, and allow for evidence-based practice. Many pediatric RCTs are published annually in high impact journals, however, outcome measures in these trials may not be valid or consistently reported [1-3]. If the outcome measures used in clinical trials are not valid, the results of the trials themselves are questionable. Indeed, much heterogeneity in outcome selection and reporting has been observed amongst clinical trials of specific diseases [4,5].

In an effort to address the issue of outcomes reporting in pediatric trials, an interdisciplinary team of researchers at the University of Alberta developed the PORTal (Primary Outcomes Reporting in Trials) initiative. In collaboration with COMET and StaRChild Health, PORTal plans to develop a database of validated pediatric outcome measures that can be accessed by child health researchers. Outcomes that should be measured and reported in all clinical trials of a specific condition, regardless of statistical significance, would also help reduce the problem of outcome reporting bias [6]. Uniform selection of outcomes would make interpretation of results and comparison across trials simpler, making meta-analyses more feasible [4].

According to a 2011 systematic review by Sinha et al., very few studies have addressed the appropriate selection of outcomes for clinical research involving children [7]. This review also identified 13 conditions for which some work has already been done to determine which outcomes should be measured in pediatric clinical trials. Since anaphylaxis was not amongst those conditions, an assessment of the heterogeneity and quality of reporting of outcome measures was considered useful.

Anaphylaxis is a serious and potentially fatal allergic reaction with a rapid onset [8]. In 2005, an expert panel published a set of three criteria defining anaphylaxis [8]. The three criteria are: 1. Acute onset of illness with involvement of skin, mucosal tissue, or both, AND at least one other system involved (respiratory compromise, OR cardiovascular compromise/associated end-organ dysfunction); 2. Two or more of: skin-mucosal, respiratory, reduced BP/associated end-organ dysfunction, gastrointestinal symptoms, occurring rapidly after exposure to a likely allergen for that patient; 3. Reduced blood pressure minutes to hours after exposure to a known allergen for that patient. According to their consensus, anaphylaxis is highly likely when any one of these three criteria is fulfilled.

Anaphylaxis can be triggered by food, insect venom, medication, latex, exercise, or unknown causes [9-16]. Regardless of the inciting cause, the final common pathway is release of histamine and other mediators from mast cells and basophils. Anaphylaxis may be fatal within minutes, usually through cardiovascular or respiratory compromise, or both [17-23]. Biphasic reactions, defined as a recurrence of anaphylactic symptoms after initial resolution, can occur 1 h to 72 h after the initial onset of symptoms [24].

In addition to epinephrine, H1-receptor antagonists and H2-receptor antagonists (i.e. antihistamines such as cetirizine and ranitidine, respectively), and corticosteroids are often used in acute therapy and after discharge. Most experts recommend these additional therapies despite limited data to support their use since these drugs are thought to be unlikely to cause harm and theoretically have some added benefit in the resolution of symptoms [23].

The most recent systematic reviews of anaphylaxis treatment, published in the Cochrane database, indicate an alarming paucity of high quality evidence supporting currently accepted treatments, including epinephrine, glucocorticoids, antihistamines and supportive care (e.g. oxygen, fluid resuscitation, raising legs above the head, inhaled bronchodilators) [25-27].

The present systematic review was planned to identify and assess the outcome measures used in RCTs of anaphylaxis treatment in children. As a secondary objective, this systematic review would assess the evidence for current treatment modalities for pediatric anaphylaxis.

## Methods

### Data sources

The search strategy was developed in conjunction with a clinical librarian. The following electronic bibliographic databases were searched: MEDLINE, EMBASE, The Cochrane Library and Cochrane Central Register of Controlled Trials (CENTRAL), and CINAHL.

The search strategy included all terms relating to the condition (anaphylaxis). The terms were combined with the Cochrane MEDLINE filter for controlled trials of interventions, and pediatrics (children/infants/adolescents). The search strategy for MEDLINE is available in the Appendix. The search terms were adapted for use with other bibliographic databases in combination with database-specific filters, where these were available. The search was limited to English-language studies, and studies published between January 2001 and December 31, 2012, in order to assess for improved quality of outcome reporting in studies published post-CONSORT (Consolidated Standards of Reporting Trials) guidelines [28].

The following websites were searched for ongoing/registered clinical trials on the topic: https://portal.nihr.ac.uk/Pages/NRRArchive.aspx; http://clinicaltrials.gov/; http://www.controlled-trials.com; http://www.anzctr.org.au/.

### Study selection

Two reviewers (TR, JC) independently screened titles and abstracts of identified references. Both reviewers also independently searched the websites for ongoing trials. Studies were included if they were a. RCTs; b. involved pediatric patients (0-18 years of age inclusive); c. investigated anaphylactic reactions from any triggering cause (including food, insect venom, medication, biologic, diagnostic agent, vaccinations, latex, exercise, or idiopathic cause) and; d. compared any acute treatment of anaphylaxis (pharmacologic, supportive measures) with any control treatment (including, but not limited to placebo). Any modality studied for the acute treatment of anaphylaxis was considered, including: epinephrine of any dose, timing and mode of administration; glucocorticoids of any dose, timing and mode of administration; inhaled beta-2 agonists; antihistamines (H1 and/or H2 antihistamines); novel treatments; and observation/ supportive care by skilled professionals in a healthcare setting. Studies focusing on the prevention of anaphylaxis (e.g. by immunotherapy) were excluded. Any and all outcome measures used in current research of pediatric anaphylaxis were included.

## Results

**Figure 1:**
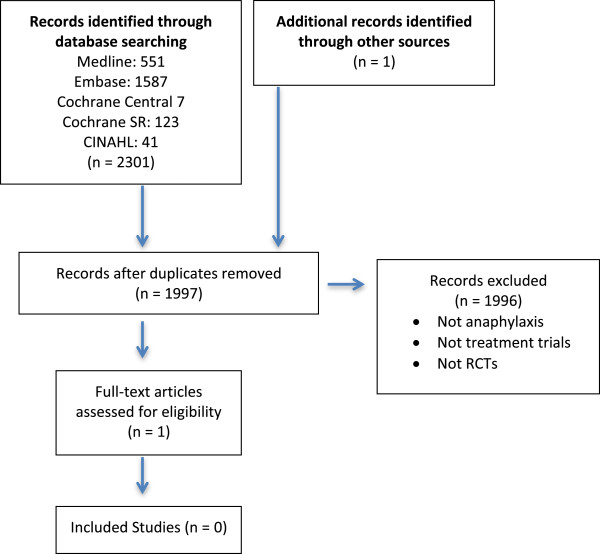
Search flow diagram.

In total, our combined searches yielded 1996 citations (see Figure 1). After screening, no studies were identified that met all inclusion criteria. The vast majority of references were not articles primarily relating to anaphylaxis, or anaphylaxis treatment. References relating to specifically to anaphylaxis treatment were reviews, systematic reviews, case reports, case series, and other types of observational (usually retrospective) studies. There were a number of controlled trials relating to anaphylaxis prevention, but none about treatment of pediatric anaphylaxis.

The search of the UK National Research Register, Current Controlled Trials, and Clinical Trials using anaphylaxis as a keyword identified no useful proposed, ongoing, or completed studies.

One study registered in the Australia/New Zealand Clinical Trials Registry in March 2011 met inclusion criteria. This was an RCT of intravenous versus intramuscular (control) epinephrine treatment of acute anaphylaxis in an emergency department in patients 15 years of age and older. The status of the trial in the database indicated that the investigators were not yet recruiting as of April 2011. Attempts to contact the primary investigator via email were unsuccessful [29].

One additional study identified through the search was an RCT comparing the sedative properties of cetirizine versus diphenhydramine in the treatment of acute food-induced allergic reactions in children [30]. The full text of this article was extracted and reviewed and, ultimately, excluded because the condition being studied was not anaphylaxis, but rather all allergic reactions.

## Discussion

This review failed to uncover any completed randomized or controlled trials of pediatric anaphylaxis treatments despite a broad search strategy. Therefore, the primary objective could not be determined. In the one planned RCT of IV versus IM epinephrine, the two main outcomes proposed were resolution of the main clinical features of anaphylaxis (defined according to consensus definition), [29], or improvement on an ordinal severity scale at 15 minutes. The planned secondary outcome was adverse effects at 15 and 60 minutes. The definition of anaphylaxis and its resolution were current, and the outcome measures proposed are appropriate.

### Clinical implications

Epinephrine remains the treatment of choice for anaphylaxis although there are no RCTs supporting its use. Epinephrine has been relatively well investigated in both children and adults in observational studies [31], RCTs involving non-anaphylactic patients [32-35], epidemiologic studies [9,36,37], fatality studies [18,19,38], and in vitro and animal studies [22,39]. Many of these studies have identified that delays in instituting treatment with epinephrine are associated with risks of mortality [40-42].

On the basis of this evidence, an expert panel published the 2011 National Institute of Allergy and Infectious Diseases (NIAID) guidelines for acute treatment of anaphylaxis. They recommended immediate use of intramuscular epinephrine, concluding that the benefits far outweigh the risks [43]. They concluded that the quality of evidence is moderate, although the contribution of expert opinion is still significant. NIAID dosing recommendations for Epinephrine 1:1000 solution for children and adults is based on pharmacologic studies. They recommend 0.15 mg for children weighing 10-25 kg, and 0.3 mg for children over 25 kg. They also suggest repeat dosing every 5-15 minutes as needed.

While second line medications such as corticosteroids and antihistamines are still recommended as adjunctive treatments, the expert panel did note the lack of evidence for these modalities. Possible reasons for continuing poorly investigated interventions in pediatrics include: “biological plausibility” of the intervention, extrapolation of data from adult studies, acceptance of lower quality research, lack of known significant harm of the intervention, and a need to do “something” rather than nothing in acute situations. Perceived concerns about medico-legal liability may also contribute to provision of treatments with no known benefits and lack of perceived harm. In their report, NIAID identified several knowledge gaps, including the role of adjunctive treatments for anaphylaxis (steroids, antihistamines and others), appropriate treatment of biphasic or protracted reactions, and the benefits and risks of alternative routes of epinephrine dosing (e.g. sublingual).

Studies have found that, despite epinephrine being the only anaphylaxis treatment with demonstrated efficacy in preventing mortality, it is not used consistently, and second-line or adjunctive medications are more often administered, sometimes before epinephrine. For example, a retrospective cross-sectional study of patients seen in a pediatric emergency department over a 5-year period with a final diagnosis of anaphylaxis showed a rate of epinephrine administration of only 54%. This was less than the rate of corticosteroids (78%) and H1 and/or H2 receptor antagonists (92%) [44].

The administration of adjunctive medications can potentially delay the use of other, perhaps more effective treatment modalities and therefore might contribute to morbidity and mortality. Furthermore, both antihistamines and steroids have adverse effects and costs, and may theoretically mask important markers of ongoing anaphylaxis risk. Recurrent dosing of both epinephrine and steroids in the emergency department also carries risks of systemic side effects. These factors need to be weighed against the potential benefit of these treatments.

### Limitations

A limitation of the current systematic review is that it was limited to studies done in the last twelve years. This time frame was considered acceptable as there are recent systematic reviews investigating anaphylaxis treatments that found no RCTs in databases extending up to thirty years into the past. Further limitations include the exclusion of non-English and non-registered trials.

### Research implications

There are a number of possibilities to explain the current gap in research. Firstly, there are clearly ethical issues involved in obtaining informed consent (or deferring consent) in emergency situations. Secondly, current anaphylaxis treatments are usually life saving, generally safe, and well established, contributing to the perceived lack of relevance of specific questions regarding pharmacotherapy. Thirdly, due to the clinical nature of anaphylaxis diagnosis, lack of accepted standards for determining degree of severity [45,46] and lack of objective point of care testing, study design can be challenging. Fourthly, a large number of patients is required for an adequately powered study assessing an uncommon condition such as pediatric anaphylaxis, and would require considerable resources over a long period of time. Additionally, prospectively collected data is essential and more valid in establishing accurate rates of adverse effects of pharmacotherapy and other interventions, but is also costly. Finally, pediatric research is associated with additional challenges, including a vulnerable and smaller population, the challenge of obtaining informed consent/assent from children, and the need to address family-centered outcomes in both treatment and investigation.

Institutional review boards serve to protect the public from the harms of unethical research, but must balance this responsibility against the imperative of advancing medical care through high quality investigation. Emergency situations, when consent cannot be obtained readily without compromising patient care, pose a particular challenge for researchers. Nevertheless, resuscitation research, which by its nature must be studied in emergency situations, is absolutely necessary, and ultimately benefits patient care.

Multiple organizations around the world have recognized the importance of resuscitation research, and the challenges of obtaining individual informed consent in this context. Therefore, guidelines for ethical research involving institutional (or deferred) consent have been developed. For example, a research ethics board may allow research that involves medical emergencies to be carried out without the consent of participants, or of their authorized third party, if a number of conditions are met [47-49]. For example, a waiver of consent may be obtained if: a. the research involves no more than minimal risk to the subjects; b. the waiver or alteration will not adversely affect the rights and welfare of the subjects; c. the research could not practicably be carried out without the waiver; d. whenever appropriate, the subjects are provided with additional pertinent information after participation [49].

Although rare, placebo-controlled trials done in emergency situations exist [50,51]. It is certainly possible to design trials studying anaphylaxis in children that meet our criteria. For example, while withholding a known lifesaving treatment, such as epinephrine, would clearly be unethical, conducting a trial to determine the optimal dosing interval or route of administration, would be unlikely to involve greater risk to the patient if the control is the standard of care (intramuscular epinephrine). In this situation, the research also offers a possibility of direct benefit to the participant. A placebo-controlled trial of antihistamines, on the other hand, could be both ethical and feasible.

There are numerous potential outcome measures that should be used and reported in all future research of anaphylaxis treatment in children. Selecting valid and clinically relevant outcomes is of paramount importance. Potential outcome measures include: mortality rate; incidence of biphasic reaction and prolonged anaphylaxis; rates of interventions other than study drug (e.g. beta agonists, steroids, antihistamines, non-epinephrine vasoactive drugs); hospitalization rate; length of emergency department visit; length of hospital stay; rate of re-presentation to hospital; pediatric specific measures (e.g. pediatric health/mental health scales); and incidence of any adverse events reported for any treatment delivered. The design and validation of a universal anaphylaxis severity rating scale could be of potential benefit, and may offer a useful outcome measure.

Future research in anaphylaxis should also collect information regarding discharge practices for cases of anaphylaxis, including prescriptions for injectable epinephrine devices, advice for optimization of asthma if relevant, referral to allergists, and anaphylaxis education.

## Conclusions

There is an alarming absence of RCTs evaluating the treatments for anaphylaxis in children. High quality studies are needed and are possible to design despite the severe and acute nature of this condition. Future trials should ensure appropriate comparator therapy that satisfies the ethical constraints inherent in resuscitation research and studies involving children, and the selection of appropriate outcome measures.

Research designs other than RCTs are still useful and can enhance the evidence for anaphylaxis treatment. Practice surveys in different settings, observational studies, and qualitative patient/family-centered studies are feasible and easy to design. Based on the results of this systematic review, it is impossible to make conclusive recommendations regarding optimal dosing, timing, or mode of administration of epinephrine for anaphylaxis. Furthermore, there is no evidence from RCTs supporting or refuting the use of common adjunctive treatments, such as corticosteroids and antihistamines, in the acute treatment of anaphylaxis, in preventing biphasic reactions, or after discharge from the ER. Until high-quality studies are designed and executed, current practices and guidelines on the management of anaphylaxis will continue to be based on lower quality evidence and on the consensus opinions of experts.

The careful selection and validation of outcome measures for future studies on pediatric anaphylaxis will support meaningful research and better treatment of children with this condition. Consistent universal reporting of these outcomes will protect against outcome reporting bias.

## Appendix

### **Cochrane SR**

1. anaphylaxis.mp. [mp=title, short title, abstract, full text, keywords, caption text]

2. anaphylactic shock.mp. [mp=title, short title, abstract, full text, keywords, caption text]

3. anaphyla*.mp. [mp=title, short title, abstract, full text, keywords, caption text]

4. systemic anaphylaxis.mp. [mp=title, short title, abstract, full text, keywords, caption text]

5. idiopathic anaphylaxis.mp. [mp=title, short title, abstract, full text, keywords, caption text]

6. 1 or 2 or 3 or 4 or 5

7. (child* or infan* or adolescen*).mp. [mp=title, short title, abstract, full text, keywords, caption text]

8. 6 and 7

9. limit 8 to last 10 years

### **Cochrane Central**

1. exp Anaphylaxis/

2. anaphylactic shock.mp. [mp=title, original title, abstract, mesh headings, heading words, keyword]

3. anaphylact*.mp. [mp=title, original title, abstract, mesh headings, heading words, keyword]

4. anaphylax*.mp. [mp=title, original title, abstract, mesh headings, heading words, keyword]

5. acute systemic allergic react*.mp. [mp=title, original title, abstract, mesh headings, heading words, keyword]

6. (acute adj3 allerg*).mp. [mp=title, original title, abstract, mesh headings, heading words, keyword]

7. idiopathic anaphylaxis.mp. [mp=title, original title, abstract, mesh headings, heading words, keyword]

8. systemic anaphylaxis.mp. [mp=title, original title, abstract, mesh headings, heading words, keyword]

9. 1 or 2 or 3 or 4 or 5 or 6 or 7 or 8

10. (child* or infan* or adolescen*).mp. [mp=title, original title, abstract, mesh headings, heading words, keyword]

11. 9 and 10

12. limit 11 to yr="2001 - 2011"

13. limit 12 to randomized controlled trial

### **CINAHL**

S1 TX randomized controlled trail

S2 TX Clinical trials

S3 AB trial

S4 AB randomly

S5 AB placebo

S6 AB randomi?ed

S7 AB groups

S8 S1 or S2 or S3 or S4 or S5 or S6 or S7

S9 MW Animals

S10 MW Human

S11 9 not (9 and 10)

S12 8 not 11

S13 MW Anaphylaxis

S14 MW Anaphylactic shock

S15 TX anaphylact*

S16 TX anaphylax*

S17 TX "acute systemic allergic react*"

S18 TX acute adj3 allerg*

S19 TX idiopathic anaphylaxis

S20 TX systemic anaphylaxis

S21 S13 or S14 or S15 or S16 or S17 or S18 or S19 or S20

S22 (S13 or S14 or S15 or S16 or S17 or S18 or S19 or S20) and (S12 and S21)

S24 Narrow by SubjectAge: - Infant: 1-23 months

Narrow by SubjectAge: - Child, Preschool: 2-5 years

Narrow by SubjectAge: - Adolescent: 13-18 years

Narrow by SubjectAge: - Child: 6-12 years

S25 Limiters - Published Date from: 20010101-20111231

S26 Limiters - English Language

### **Embase**

1. randomized controlled trial/

2. clinical trial/

3. randomi?ed.ti,ab.

4. placebo.ti,ab.

5. dt.fs.

6. randomly.ti,ab.

7. trial.ti,ab.

8. groups.ti,ab.

9. 1 or 2 or 3 or 4 or 5 or 6 or 7 or 8

10. animal/

11. human/

12. 10 not (10 and 11)

13. 9 not 12

14. anaphylactic shock.mp. or exp anaphylactic shock/

15. anaphylact*.mp. [mp=title, abstract, subject headings, heading word, drug trade name, original title, device manufacturer, drug manufacturer, device trade name, keyword]

16. anaphylax*.mp. [mp=title, abstract, subject headings, heading word, drug trade name, original title, device manufacturer, drug manufacturer, device trade name, keyword]

17. acute systemic allergic react*.mp. [mp=title, abstract, subject headings, heading word, drug trade name, original title, device manufacturer, drug manufacturer, device trade name, keyword]

18. (acute adj3 allerg*).mp. [mp=title, abstract, subject headings, heading word, drug trade name, original title, device manufacturer, drug manufacturer, device trade name, keyword]

19. idiopathic anaphylaxis.mp. [mp=title, abstract, subject headings, heading word, drug trade name, original title, device manufacturer, drug manufacturer, device trade name, keyword]

20. systemic anaphylaxis.mp. [mp=title, abstract, subject headings, heading word, drug trade name, original title, device manufacturer, drug manufacturer, device trade name, keyword]

21. 14 or 15 or 16 or 17 or 18 or 19 or 20

22. 13 and 21

23. (infan* or child* or adolescen*).ti,ab,kw,tw.

24. 22 and 23

25. limit 24 to english language

26. limit 25 to yr="2001 - 2011"

### **Medline**

1. randomized controlled trial/

2. clinical trial.pt.

3. randomi?ed.ti,ab.

4. placebo.ti,ab.

5. dt.fs.

6. randomly.ti,ab.

7. trial.ti,ab.

8. groups.ti,ab.

9. or/1-8

10. Animals/

11. Humans/

12. 10 not (10 and 11)

13. 9 not 12

14. anaphylactic shock.mp. or exp Anaphylaxis/

15. anaphylact*.mp. [mp=protocol supplementary concept, rare disease supplementary concept, title, original title, abstract, name of substance word, subject heading word, unique identifier]

16. anaphylax*.mp. [mp=protocol supplementary concept, rare disease supplementary concept, title, original title, abstract, name of substance word, subject heading word, unique identifier]

17. acute systemic allergic react*.mp. [mp=protocol supplementary concept, rare disease supplementary concept, title, original title, abstract, name of substance word, subject heading word, unique identifier]

18. (acute adj3 allerg*).mp. [mp=protocol supplementary concept, rare disease supplementary concept, title, original title, abstract, name of substance word, subject heading word, unique identifier]

19. idiopathic anaphylaxis.mp. [mp=protocol supplementary concept, rare disease supplementary concept, title, original title, abstract, name of substance word, subject heading word, unique identifier]

20. systemic anaphylaxis.mp. [mp=protocol supplementary concept, rare disease supplementary concept, title, original title, abstract, name of substance word, subject heading word, unique identifier]

21. 14 or 15 or 16 or 17 or 18 or 19 or 20

22. 13 and 21

23. limit 22 to "all child (0 to 18 years)"

24. limit 23 to english language

25. limit 24 to yr="2001 - 2011"

## Competing interests

The authors declare that they have no competing interests.

## Authors’ contributions

TR conceptualized and designed the systematic review, drafted the initial manuscript, and approved the final manuscript as submitted. JC acted as a second reviewer for the systematic review, reviewed and revised the manuscript, and approved the final manuscript as submitted. DA critically reviewed the manuscript, and approved the final manuscript as submitted. HJ critically reviewed the manuscript and approved the final manuscript as submitted. SV helped to conceptualize this systematic review, critically reviewed the manuscript, and approved the final manuscript as submitted.

## Pre-publication history

The pre-publication history for this paper can be accessed here:

http://www.biomedcentral.com/1471-2431/14/158/prepub
